# Sterblichkeit der Lungenembolie in der DACH-Region

**DOI:** 10.1007/s00063-021-00854-9

**Published:** 2021-08-24

**Authors:** Lukas Hobohm, Tim Sebastian, Luca Valerio, Seyed Hamidreza Mahmoudpour, Georgios Vatsakis, Fabian Johner, Karsten Keller, Thomas Münzel, Nils Kucher, Stavros V. Konstantinides, Stefano Barco

**Affiliations:** 1 Centrum für Thrombose und Hämostase (CTH), Universitätsmedizin Mainz, Langenbeckstraße 1, 55131 Mainz, Deutschland; 2 Zentrum für Kardiologie, Universitätsmedizin Mainz, Mainz, Deutschland; 3 Klinik für Angiologie, Universitätsspital Zürich, Zürich, Schweiz; 4 Institut für Medizinische Biometrie, Epidemiologie und Informatik (IMBEI), Universitätsmedizin Mainz, Mainz, Deutschland; 5 Innere Medizin VII, Universität Heidelberg, Heidelberg, Deutschland

**Keywords:** Prävention, Therapie, Deutschland, Österreich, Schweiz, Prevention, Treatment, Germany, Austria, Switzerland

## Abstract

**Hintergrund:**

Kürzlich veröffentliche Studien zeigen eine steigende Inzidenz für die Lungenarterienembolie (LE) bei gleichzeitigem Rückgangs der LE-assoziierten Mortalität.

**Ziel der Studie:**

Detaillierte Daten zur Mortalität der LE in Deutschland, Österreich und der Schweiz (DACH-Region) sind derzeit nicht vorhanden.

**Material und Methoden:**

Datensätze wurden aus der Mortalitätsdatenbank der Weltgesundheitsorganisation (WHO) ausgewertet. Hierbei analysierten wir die Häufigkeit sowohl der akuten LE als auch der tiefen/oberflächlichen Venenthrombose als primärer Todesursache.

**Ergebnisse:**

Demnach sank die jährliche altersstandardisierte Mortalität zwischen Januar 2000 und Dezember 2015 von 15,6 auf 7,8 Todesfälle pro 1000 Einwohner. Zwischen Januar 2012 und Dezember 2016 ereigneten sich in der DACH-Region (Bevölkerungsanzahl: 98.273.320 Menschen) durchschnittlich 9127 durch LE verursache Todesfälle pro Jahr. Interessanterweise ist LE–assoziierte Gesamtmortalität bei Frauen zwischen dem 15. und 55. Lebensjahr deutlich höher als bei gleichaltrigen Männern.

**Schlussfolgerung:**

Der Rückgang der Mortalität durch die Erkrankung LE seit dem Jahr 2000 ist vermutlich durch eine verbesserte Patientenversorgung mit Einführung neuer Antikoagulanzien und durch den vermehrten Einsatz und diagnostischen Fortschritt bei den computertomographischen Untersuchungen erklärt. Festzuhalten ist, dass die LE eine wichtige Todesursache vor allem im höheren Alter darstellt. Außerdem ist der Anteil der Frauen im gebärfähigen Alter, die nach einer akuten LE sterben, mit 3,5 % hoch. Daher sind, trotz des medizinischen Fortschritts, weitere Anstrengungen für eine Verbesserung der Prävention, Diagnostik und Therapie, aber insbesondere auch des Krankheitsbewusstseins notwendig.

## Abstract (en)

**Background:**

Pulmonary embolism (PE)-related mortality is decreasing worldwide.

**Aim:**

Little is known about the burden imposed by pulmonary embolism for Germany, Austria and Switzerland (DACH countries).

**Materials and methods:**

We aimed to assess pulmonary embolism-related mortality and time trends for the DACH countries based on data from the WHO Mortality Database. Deaths were considered pulmonary embolism-related if the International Classification of Disease-10 code for acute pulmonary embolism or any code for deep or superficial vein thrombosis was listed as the primary cause of death.

**Results:**

Between 2000 and 2015, age-standardized annual pulmonary embolism-related mortality rates decreased linearly from 15.6 to 7.8 deaths per 1000 population. In the 5‑year period between 2012 and 2016, an average of 9127 pulmonary embolism-related deaths occurred annually in the DACH countries with a population of 98,273,329. Interestingly, pulmonary embolism-related mortality rates were considerably higher among women aged 15–55 years compared to age-matched men.

**Conclusion:**

The observed decreasing trends in pulmonary embolism-related mortality might reflect improved management of the disease including new treatment options as well as advances in imaging technologies. However, pulmonary embolism remains a substantial contributor to total mortality, especially among women aged 15–55 years. For this reason, campaigns to increase physician and public awareness are urgently required to further improve the management and treatment of this preventable thrombotic disorder, which still remains the leading preventable cause of death.

## Hintergrund und Fragestellung

Die venöse Thromboembolie (VTE), die die Entitäten der tiefen Beinvenenthrombose (TVT) und der akuten Lungenarterienembolie (LE) umfasst, stellt eine häufige akute kardiovaskuläre Erkrankung dar. Die akute LE gilt nach dem Myokardinfarkt und dem Schlaganfall als die dritthäufigste kardiovaskuläre Erkrankung mit einer jährlichen Inzidenzrate von 39–115 neuen Fällen pro 100.000 Einwohner [5, 6, 17]. Innerhalb der letzten zwei Jahrzehnte wurde in vielen westlichen Ländern eine Zunahme der Inzidenz und Abnahme der Letalität beobachtet [4, 11, 12]. Auch wenn die Gesamtzahl der Todesfälle nach einer LE relativ gering erscheint, liegt sie weiterhin bei mehr als 80 Todesfälle pro 100.000 Einwohner unter älteren Menschen, die älter als 80 Jahre waren. Bei jüngeren Menschen ist zwar die Mortalität niedriger, allerdings ist die akute LE – insbesondere bei Frauen im Alter von 15–55 Jahren – im Vergleich zu anderen Erkrankungen eine relativ häufige Todesursache und für bis zu 13 von 1000 Todesfällen verantwortlich [3].

Detaillierte Daten zur Entwicklung der LE-assoziierte Mortalität aus der DACH-Region (Deutschland, Österreich, Schweiz) fehlen. Die Mortalität nach einer LE war zuletzt bis 2004 in Deutschland gestiegen, während sie in Österreich leicht gesunken war [10]. Neueste Untersuchungen zeigten, dass zwischen den Jahren 2005 und 2015 die Hospitalisierungen aufgrund akuter LE-Ereignisse in Deutschland anstiegen bei gleichzeitiger Abnahme der Mortalität [11]. Ein ähnlicher Trend wurde in einer Auswertung von Versicherungsdaten der AOK Hessen aus den Jahren 2000 bis 2006 beobachtet [13].

Das Ziel dieser Arbeit war die Entwicklung der lungenembolieassoziierten Mortalität in der DACH-Region innerhalb der letzten zwei Jahrzehnte anhand validierter Personendaten aus der Mortalitätsdatenbank der Weltgesundheitsorganisation (WHO) altersstandardisiert zu untersuchen.

## Studiendesign und Untersuchungsmethoden

Die Mortalitätsdatenbank der WHO beinhaltet Datensätze zur primären Todesursache aller erfassten Todesfälle aus den jeweiligen Mitgliedsstaaten, welche jährlich an die WHO gemeldet werden und nach Alter und Geschlecht untergliedert übermittelt werden. Die primäre Todesursache ist dabei als die Erkrankung oder das Ereignis definiert, welche oder welches unmittelbar zum Tod führte. Diese Daten stammen aus Registern der nationalen Standesämter, welche gemeldete Todesfälle anhand der internationalen statistischen Klassifikation der Krankheiten und verwandter Gesundheitsprobleme nach ICD-10 klassifizieren. Die WHO führt nach standardisiertem Vorgehen vor Veröffentlichung dieser Daten eine Plausibilitätsprüfung durch.

In dieser vorliegenden Arbeit wurden die Zahl der mit LE assoziierten Todesfälle, die Gesamttodesfälle und die Bevölkerungszahlen aus der WHO-Datenbank für das Zeitfenster der Jahre 2000 bis 2016 analysiert (Stand 05/2019); einschränkend bleibt zu bemerken, dass für Österreich die oben genannten Daten erst ab dem Jahr 2002 verfügbar waren. Die zeitliche Entwicklung der berechneten Mortalitätsraten wurden für die verschiedenen Alters‑, Geschlechts- und Ländergruppen analysiert und werden im Folgenden beschrieben.

Dabei wurden Todesfälle ursächlich einer LE zugeschrieben, wenn:

1. die primäre Todesursache als akute LE mit oder ohne Angabe eines Cor pulmonale kodiert wurde (I26);
2. oder ein Code verwendet wurde, welcher einer sonst nicht tödlichen Manifestation einer venösen Thromboembolie zuzuordnen ist; dies beinhaltet beispielsweise eine tiefe Beinvenenthrombose, Phlebitis und Thrombophlebitis (I80).

Dieses Vorgehen wurde bereits bei einer früher publizierten Veröffentlichung über die europäischen Trends der LE-assoziierten Mortalität angewandt und kann dort im Detail nachgelesen werden.

Die LE-verbundene Mortalität („crude mortality rate“) wurde berechnet, indem die Anzahl der durch LE verursachten Todesfälle durch die Bevölkerungszahl (angegeben pro 100.000 Personen) im entsprechenden Zeitintervall dividiert wurde. Der Anteil der LE an der Gesamtmortalität („proportional mortality rate“) wurde berechnet, indem die Anzahl der durch LE verursachten Todesfälle durch die Anzahl aller Todesfälle im entsprechenden Zeitintervall dividiert wurde (angegeben pro 1000 Personen). Diese Berechnungen erfolgten sowohl für die Periode 2012 bis 2016 als auch für die individuellen Jahre zwischen 2000 und 2016. Der Zeitraum 2012 bis 2016 wurde gewählt, um gesondert nochmals aktuelle Mortalitätsdaten zu analysieren.

Um einen geografischen und zeitlichen Vergleich in den DACH-Ländern und Westeuropa (Belgien, Deutschland, Frankreich, Luxemburg, Niederlande, Österreich, Schweiz) zu ermöglichen, erfolgte eine Altersstandardisierung der Mortalität unter Angabe des 95%-Konfidenzintervalls. Dazu verwendeten wir die europäische Standardpopulation, welche durch die Europäische Kommission 2013 veröffentlich wurde [16]. Die Datensätze wurden dabei in 18 Gruppen à 5-Jahres-Altersklassen (0–4 Jahre, 5–9 Jahre, 10–14 Jahre, 15–19 Jahre, 20–24 Jahre, 25–29 Jahre, 30–34 Jahre, 35–39 Jahre, 40–44 Jahre, 45–49 Jahre, 50–54 Jahre, 55–59 Jahre, 60–64 Jahre, 65–69 Jahre, 70–74 Jahre, 75–79 Jahre, 80–84 Jahre, > 85 Jahre) unterteilt.

Um mögliche Veränderungen der altersstandardisierten Mortalität der LE über die Zeit (*Trendanalysen*) zu untersuchen, wurden eine Joinpoint-Regressionsanalyse (JoinPoint Version 4.6.0.0) nach dem Geschlecht durchgeführt. Dieses Modell identifiziert anhand des Regressionsgraphen Änderungspunkte („joinpoints“) und liefert die dazugehörigen durchschnittlichen Änderungsraten pro Zeitabschnitt, welche als Steigungen („slopes“) mit dem dazugehörigen 95%-Konfidenzintervall (KI) angegeben werden.

## Ergebnisse

Zwischen 2002 und 2016 wuchs die Gesamtbevölkerung der DACH-Region von 97,9 auf 99,5 Mio. Einwohner (Deutschland: 82,5–82,3 Mio.; Österreich: 8,1–8,7 Mio.; Schweiz: 7,3–8,4 Mio.). In diesem Zeitraum war die LE regional für insgesamt 157.760 Todesfälle verantwortlich (Deutschland: 143.145; Österreich: 8459; Schweiz: 6156). Der Anteil der LE an der Gesamtmortalität in der DACH-Region sank im Beobachtungszeitraum von 13,0 pro 1000 Todesfällen im Jahr 2000 auf 8,1 pro 1000 Todesfällen im Jahr 2016; für Frauen von 14,9 auf 9,0 pro 1000 Todesfällen und für Männer von 10,8 auf 7,1 pro 1000 Todesfällen.

**Figure 1:**
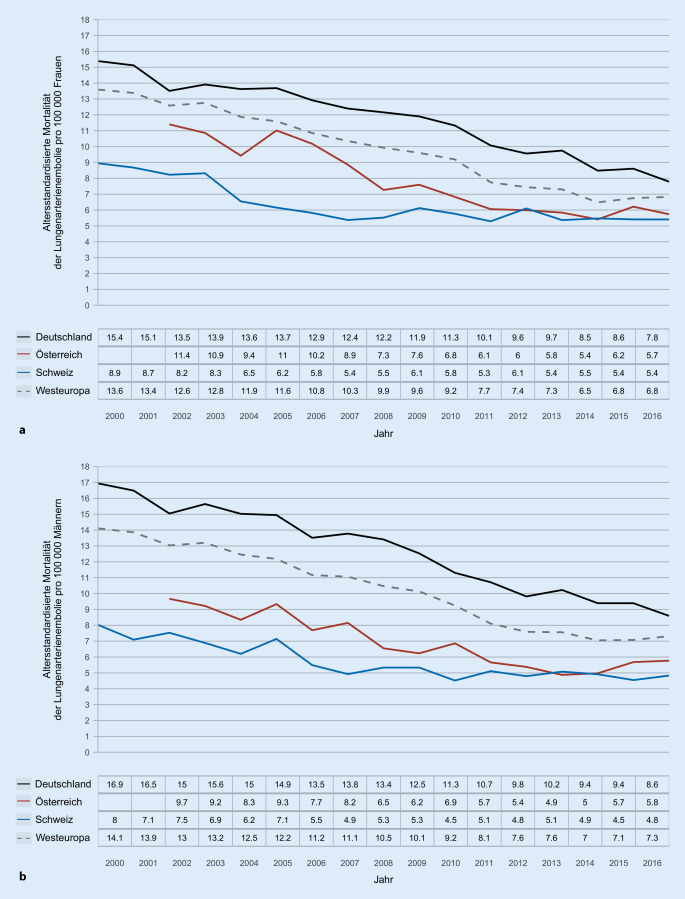

**Figure 2:**
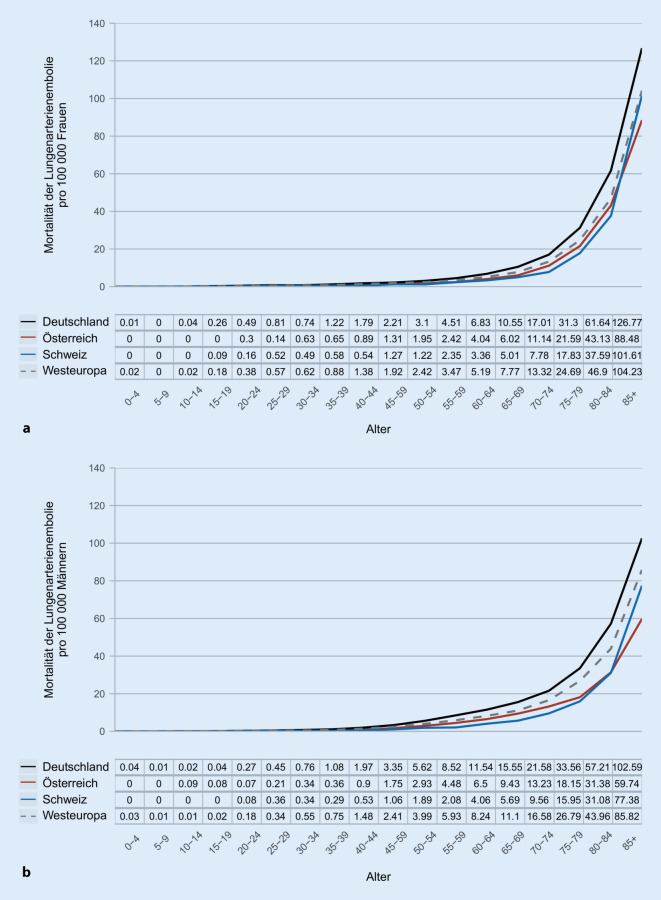

Die durchschnittliche Gesamtbevölkerung in der DACH-Region betrug 98,3 Mio. Personen zwischen den Jahren 2012 und 2016. Innerhalb dieser fünfjährigen Periode wurden 45.635 Todesfälle der LE zugeordnet. Diese verteilten sich auf 41.151 Fälle in Deutschland, 2403 Fälle in Österreich und 2081 Fälle in der Schweiz. Die Mortalität an Lungenembolie stieg mit zunehmendem Alter bei Frauen (Abb. 1a) sowie bei Männern (Abb. 1b) exponentiell an. Im gleichen Beobachtungszeitraum war der Anteil der LE an der Gesamtmortalität bei Frauen zwischen dem 15. und 55. Lebensjahr im Vergleich zu Männern der gleichen Altersgruppe in allen Regionen deutlich erhöht (Abb. 2).

**Figure 3:**
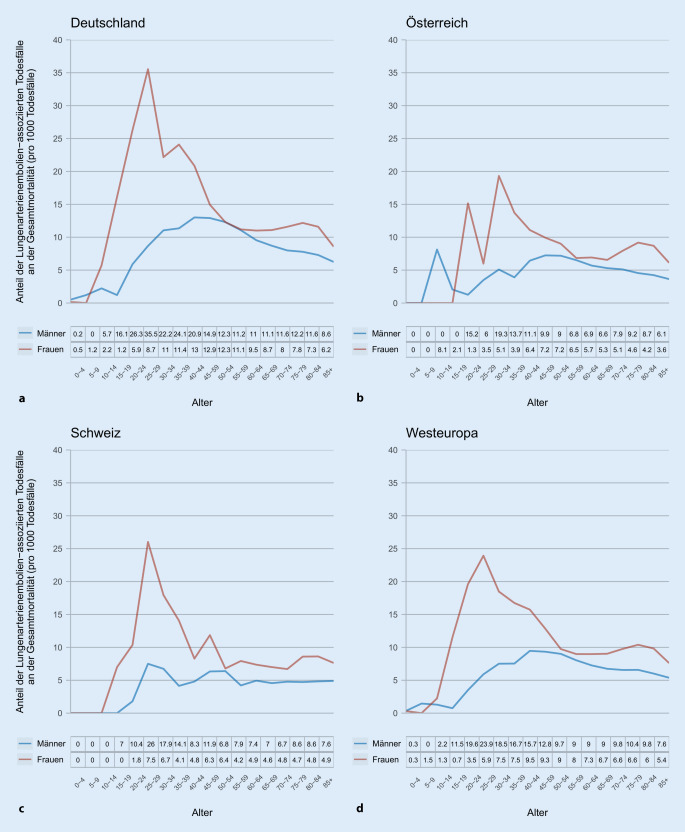

Die Mortalitätsrate der LE sank zwischen 2000 und 2016 in der DACH-Region von 13,1 auf 8,6 pro 100.000 Einwohner; für Deutschland von 13,6 auf 9,2, für Österreich von 8,8 auf 5,8 und für die Schweiz von 6,9 auf 5,1 pro 100.000 Einwohner. Die altersstandardisierte Lungenemboliemortalität nahm im Beobachtungszeitraum von 15,6 auf 7,8 pro 100.000 Einwohner ab (für Frauen von 14,9 auf 7,4 und für Männer von 16,2 auf 8,1 pro 100.000 Einwohner). Die Abb. 3 zeigt die Entwicklung der altersstandardisierten Mortalitätsrate für Frauen (Abb. 3a) und Männer (Abb. 3b) für die jeweiligen Regionen. Mit einer Mortalitätsrate von 16,2 im Jahr 2000 und 8,3 im Jahr 2016 zeigte Deutschland geschlechtsunabhängig die höchste und die Schweiz mit 8,5 in 2000 und 5,2 pro 100.000 Einwohnern im Jahr 2016 die geringste Rate der altersstandardisierten LE-Mortalität.

Für Westeuropa zeigte sich in der Joinpoint-Regressionsanalyse eine signifikante Abnahme der altersstandardisierten LE-Mortalität bei Männern um jährlich durchschnittlich 0,46 Todesfälle pro 100.000 Einwohner (95%-KI: 0,40–0,52) für den Zeitraum 2000 bis 2009 und 0,88 Todesfälle pro 100.000 Einwohner (95%-KI: 0,19–1,56) für den Zeitraum 2009 bis 2012. Ebenfalls signifikant sank die altersstandardisierte LE-Mortalität in der Gruppe der Frauen um jährlich durchschnittlich 0,52 Todesfälle pro 100.000 Einwohnern (95%-KI: 0,48–0,56) für den Zeitraum 2000 bis 2014. Ein ähnlicher Trend konnte in allen Ländern der DACH-Region beobachtet werden: In Deutschland ergab sich für den gesamten Zeitraum 2000–2016 eine durchschnittliche Abnahme von 0,53 Todesfällen pro 100.000 Einwohner (95%-KI: 0,57–0,49) bei Männern und 0,46 Todesfällen pro 100.000 Einwohner (95%-KI: 0,50–0,42) bei Frauen; in Österreich und in der Schweiz zeigte sich die Abnahme vor allem in den ersten Jahren der Periode (in Österreich, zwischen 2002 und 2013 bei Männern und zwischen 2002 und 2012 bei Frauen; in der Schweiz, zwischen 2000 und 2007 bei Männern und zwischen 2000 und 2006 bei Frauen). Die Ergebnisse der Joinpoint-Regressionsanalyse, aufgeschlüsselt nach Geschlecht und Region, finden sich in Tab. 1.

**Table 1:** 

	Männer						Frauen			
	Periode 1		Periode 2		Periode 3		Periode 1		Periode 2	
	Jahre	Slope (95%-KI)	Jahre	Slope 95%-KI	Jahre	Slope 95%-KI	Jahre	Slope 95%-KI	Jahre	Slope (95%-KI)
Österreich	2002–2013	*−0,44* *(−0,52, −0,36)*	2013–2016	0,30 (−0,39, 0,99)	–	–	2002–2012	*−0,58* *(−0,72, −0,44)*	2012–2016	0,02 (−0,57, 0,61)
Deutschland	2000–2016	*−0,53* *(−0,57, −0,49)*	–	–	–	–	2000–2016	*−0,46* *(−0,50, −0,42)*	–	–
Schweiz	2000–2007	*−0,39* *(−0,53, −0,25)*	2007–2016	−0,07 (−0,17, 0,03)	–	–	2000–2006	*−0,58* *(−0,76, −0,40)*	2006–2016	−0,03 (−0,11, 0,05)
Westeuropa*	2000–2009	*−0,46* *(−0,52, −0,40)*	2009–2012	*−0,88* *(−1,56, −0,19)*	2012–2016	−0,08 (−0,29, 0,13)	2000–2014	*−0,52* *(−0,56, −0,48)*	2014–2016	0,07 (−0,75, 0,89)

Mittels Joinpoint-Regressionsanalyse wurden Veränderungszeitpunkte („joinpoints“) identifiziert und durchschnittliche Veränderungsraten als Steigung („slope“) der Regressionsgeraden mit dem dazugehörigen 95%-Konfidenzintervall im identifizierten Zeitabschnitt angegeben. Zeitintervalle mit statistisch signifikanter Reduktion der altersstandardisierten Lungenemboliemortalität sind kursiv hervorgehoben

*KI* Konfidenzintervall

^*^Westeuropa: Belgien, Deutschland, Frankreich, Luxemburg, Niederlande, Österreich, Schweiz

## Diskussion

Die Ergebnisse dieser Arbeit bestätigen, ergänzend zu kürzlich veröffentlichten Daten der gesamteuropäischen Region, den beobachteten Rückgang der Mortalität der LE in Westeuropa und insbesondere auch in den Ländern der DACH-Region. Erklärungsansätze beinhalten Fortschritte in der Prävention, Diagnostik und Behandlung von VTE-Ereignissen. Zu den potenziellen Ursachen der gestiegenen Inzidenz und gesunken Mortalität gehört neben dem medizinischen Fortschritt mit verbessertem Überleben von Patienten mit schwerem Krankheitsverlauf auch die flächendeckende Verfügbarkeit und häufigere Anwendung von hochauflösender Schnittbildgebung der Computertomographie (CT). Durch den diagnostischen Fortschritt, insbesondere in der CT-Technik (mit höherer Sensitivität der verschiedenen Verfahren), können häufiger periphere, subsegmentale LE-Ereignisse diagnostiziert werden, welche vor dem technischen Fortschritt vermutlich nicht erkannt wurden. Daher stieg durch den diagnostischen Fortschritt die Zahl der LE-Ereignisse mit geringerer Thrombuslast und somit auch in der Mehrzahl jener mit besserer Prognose an [1, 11, 12]. Zudem resultiert hieraus aber auch zwangsläufig eine Zunahme der inzidentellen (zufälligen) Diagnose von peripheren, klinisch häufig stummen Embolien. Ein Beispiel ist die serielle Schnittbildgebung im Rahmen der Stadienbestimmung (Staging) der Patienten mit Tumorerkrankung. Es ist daher umso wichtiger zu untersuchen, ob insbesondere auch ein Rückgang der altersstandardisierten Lungenemboliemortalität zu beobachten ist. Dennoch zeigen sich in der DACH-Region deutliche länderspezifische Unterschiede.

Für Deutschland zeigte sich zwischen 2012 und 2016 die höchste (altersstandardisierte) Mortalitätsrate innerhalb der DACH-Region. Diese variierte im Beobachtungszeitraum zwischen 9,2 und 10,8 pro 100.000 Einwohnern. Damit befindet sich Deutschland oberhalb des westeuropäischen Durchschnitts. Diese jährliche Abnahme variierte im gleichen Zeitraum zwischen 8,5 und 9,9 Todesfällen pro 100.000 Einwohnern. Demgegenüber liegt die Mortalität in Österreich (5,2–6,0 Todesfälle pro 100.000 Einwohnen) und in der Schweiz (4,9–5,3 Todesfälle pro 100.000 Einwohnern) unterhalb des westeuropäischen Durchschnitts. Trotz dieser positiven Entwicklung hinsichtlich des Mortalitätsrückgangs ist und bleibt die LE eine lebensbedrohliche Erkrankung und eine häufige Todesursache; dies gilt insbesondere für Frauen zwischen dem 15. und 55. Lebensjahr.

Als mögliche Ursachen der Ungleichheit der Mortalitätsraten innerhalb der Länder der DACH-Region vermuten wir die Unterschiede in den berichteten Inzidenzen von wichtigen Risikofaktoren (wie Adipositas, ischämische Herzkrankheit und Krebserkrankungen), welche mit dem Auftreten von VTE-Ereignissen assoziiert sind. So zeigen beispielsweise Daten der Organisation für wirtschaftliche Zusammenarbeit und Entwicklung (OECD) für das Jahr 2015 in Deutschland eine fast bzw. mehr als doppelt so hohe Inzidenz einer Adipositas unter Erwachsenen (23,6 %) im Vergleich zu Österreich (14,7 %) und der Schweiz (10,3 %) [14]. Ähnlich liegt die geschätzte Inzidenz, basierend auf den Daten der Global-Burden-of-Disease-Studie (Stand 2016), für die ischämische Herzkrankheit in Deutschland mit 469 Neuerkrankungen pro 100.000 Einwohnern deutlich höher als in Österreich (375 Neuerkrankungen) und der Schweiz (351 Neuerkrankungen). Daten der Internationalen Agentur für Krebsforschung (Stand 2018) zeigen für Deutschland mit 313 Neuerkrankungen pro 100.000 Einwohnern eine höhere (altersstandardisierte) Inzidenz von Krebserkrankungen im Vergleich zu Österreich (248 Neuerkrankungen), allerdings bei einer ähnlich hohen Rate in der Schweiz (311 Neuerkrankungen) [15]. Einen alternativen, nicht krankheitsspezifischen Erklärungsansatz stellen mögliche länderspezifische Unterschiede im Kodierungsverhalten dar, wobei dies als weniger wahrscheinlich anzusehen ist.

Bisher veröffentlichte geschätzte Inzidenz- und Mortalitätszahlen für die LE müssen kritisch betrachtet und aktiv hinterfragt werden. Durch Hochrechnungen, beruhend auf einer Analyse von Cohen et al., wird die Gesamtzahl der LE-Toten in Deutschland auf 40 bis 100.000 Todesfälle pro Jahr geschätzt [7]. Diese Schätzungen basieren auf älteren Daten und der Annahme, dass die Mehrzahl (bis zu 93 %) der Todesfälle durch LE plötzlich und unerwartet auftreten oder als Folge einer nicht behandelten, nicht erkannten VTE verursacht wurden. Ob diese Annahme den aktuellen diagnostischen und therapeutischen Standards noch gerecht wird, ist als kritisch anzusehen. Dennoch ist anzumerken, dass aufgrund der hohen Dunkelziffer, die durch uns beobachtete jährliche Gesamttodeszahl von 8633 (in 2012) und 7579 (in 2016) in Deutschland, die „wahre“ LE-assoziierte Todeszahl unterschätzt wird.

Ein weiterer wichtiger Punkt für die Betrachtung der steigenden Inzidenz stellt die stetige Zunahme der CT-Untersuchungen dar. Zwischen 2007 und 2016 nahmen die CT-Untersuchungen in Deutschland um etwa 45 % zu [2]. Daten aus dem Jahr 2015 zeigen, dass in Österreich die meisten Computertomographien mit etwa 166 Untersuchungen im Jahr pro 1000 Einwohner durchgeführt werden. In Deutschland sind es 132 Untersuchungen jährlich pro 100.000 Einwohner und in der Schweiz lediglich 104 Untersuchungen jährlich pro 100.000 Einwohner. Obwohl es durch den technischen Fortschritt der Mehrschichtsspiraltomographie zu einer Zunahme der Diagnose von klinisch stummen (oft peripheren) LE gekommen ist, werden gegenteilig, durch den Rückgang der Häufigkeit von Autopsien, insbesondere, wenn kardiopulmonale Begleiterkrankungen vorliegen, tödlich verlaufende LE womöglich übersehen. Niedrige Obduktionszahlen finden sich mit 4,2 % insbesondere in der Schweiz. Im Vergleich betragen diese für die EU-Mitgliedsstaaten im Durchschnitt 15,3 % und für Österreich 11,0 %. Für Deutschland existierten keine veröffentlichten genauen Obduktionsraten von der WHO [8]. Epidemiologische Messgrößen lassen sich daher nicht zufriedenstellend erheben.

Alarmierend ist zudem die hohe LE-assoziierte Mortalität bei Frauen zwischen 15 und 55 Jahren. Eine kürzlich veröffentliche Studie zeigte, dass auch Schwangere eine hohe Mortalität aufwiesen (3–4 Tote pro 100 Schwangeren pro Jahr) und diese etwa 500-fach höher im Vergleich zu Schwangeren ohne LE ist. Bei Frauen mit LE und hämodynamischer Instabilität steigt die Krankenhausmortalität auf sehr hohe 37 % [9].

## Ausblick

Mit steigender Inzidenz stellt die LE, trotz des beobachteten Rückgangs der LE-assoziierten Mortalität, eine zunehmende Herausforderung für die Gesundheitssysteme der westlichen Länder mit alternden Gesellschaften dar. Die steigende Inzidenz von LE kann zum Teil auf den wachsenden Einsatz der CT-Untersuchungen zurückgeführt werden. Der Rückgang der LE-assoziierten Mortalität kann sowohl durch eine Verbesserung der Patientenversorgung als auch durch die stetige Zunahme an CT-Untersuchungen mit Diagnose kleinerer (bisher nicht detektierter) als auch asymptomatischer LE-Ereignisse erklärt werden. Die LE ist eine lebensbedrohliche Erkrankung und stellt eine häufige Todesursache dar; insbesondere bei Frauen zwischen dem 15. und 55. Lebensjahr ist die LE-assoziierte Mortalitätsrate im Vergleich zum männlichen Geschlecht erhöht. Die LE-assoziierten Mortalitätsraten unterscheiden sich innerhalb der Länder der DACH-Region deutlich und sind in Deutschland am höchsten. Hierfür sind vermutlich unterschiedliche Inzidenzen wichtiger Risikofaktoren wie Adipositas, ischämische Herzkrankheit und Krebserkrankungen als mögliche Ursachen der Ungleichheit der Mortalitätsraten anzuführen. Andere Erklärungsansätze beinhalten den Rückgang der Häufigkeit von Autopsien in den betreffenden Ländern und mögliche Unterschiede im Kodierungsverhalten. Gesundheitskampagnen sowie die Erforschung verbesserter Strategien zur Prävention, Diagnostik und Therapie sollten in den nächsten Jahren dazu beitragen, die Mortalität der LE weiter zu senken.

## Fazit für die Praxis

- Zwischen Januar 2000 und Dezember 2015 sank die jährliche altersstandardisierte Mortalität der akuten Lungenembolie in der DACH-Region von 15,6 auf 7,8 Todesfälle pro 1000 Einwohner.
- Die LE-assoziierte Gesamtmortalität bei Frauen zwischen dem 15. und 55. Lebensjahr war deutlich höher als bei gleichaltrigen Männern.
- Für Deutschland zeigte sich zwischen 2012 und 2016 die höchste altersstandardisierte Mortalitätsrate (zwischen 9,2 und 10,8 pro 100.000 Einwohnern) innerhalb der DACH-Region.
- Deutschland befindet sich oberhalb des westeuropäischen Durchschnitts, bezogen auf die altersstandardisierte Mortalitätsrate für die akute Lungenembolie.
- Die Erforschung von verbesserten Strategien zur Prävention, Diagnostik und Therapie sollten in den nächsten Jahren dazu beitragen, die Mortalität der LE weiter zu senken.

## References

[1] Agarwal S, Clark D, Sud K (2015). Gender disparities in outcomes and resource utilization for acute pulmonary embolism hospitalizations in the United States. Am J Cardiol.

[2] Anonymous (2020). Röntgendiagnostik: Häufigkeit und Strahlenexposition.

[3] Barco S, Mahmoudpour SH, Valerio L (2020). Trends in mortality related to pulmonary embolism in the European Region, 2000–15: analysis of vital registration data from the WHO Mortality Database. Lancet Respir Med.

[4] Barco S, Valerio L, Ageno W (2020). Age-sex specific pulmonary embolism-related mortality in the USA and Canada, 2000–18: an analysis of the WHO Mortality Database and of the CDC Multiple Cause of Death database. Lancet Respir Med.

[5] Centers for Disease Control and Prevention (CDC) (2012). Venous thromboembolism in adult hospitalizations—United States, 2007–2009. MMWR Morb Mortal Wkly Rep.

[6] Cheuk BL, Cheung GC, Cheng SW (2004). Epidemiology of venous thromboembolism in a Chinese population. Br J Surg.

[7] Cohen AT, Agnelli G, Anderson FA (2007). Venous thromboembolism (VTE) in Europe. The number of VTE events and associated morbidity and mortality. Thromb Haemost.

[8] Gateway EHI (2019). Autopsy rate (%) for all deaths.

[9] Hobohm L, Keller K, Valerio L (2020). Fatality rates and use of systemic thrombolysis in pregnant women with pulmonary embolism. ESC Heart Fail.

[10] Hoffmann B, Gross CR, Jockel KH (2010). Trends in mortality of pulmonary embolism—An international comparison. Thromb Res.

[11] Keller K, Hobohm L, Ebner M (2020). Trends in thrombolytic treatment and outcomes of acute pulmonary embolism in Germany. Eur Heart J.

[12] Konstantinides SV, Barco S, Lankeit M (2016). Management of pulmonary embolism: an update. J Am Coll Cardiol.

[13] Kroger K, Kupper-Nybelen J, Moerchel C (2012). Prevalence and economic burden of pulmonary embolism in Germany. Vasc Med.

[14] OECD (2017). Obesity update 2017.

[15] WHO (2018). Estimated age-standardized incidence rates (World) in 2018, all cancers, both sexes, all ages.

[16] Turbeville SD, Cowan LD, Owen WL (2003). Risk factors for injury in high school football players. Am J Sports Med.

[17] Wendelboe AM, Raskob GE (2016). Global burden of thrombosis: epidemiologic aspects. Circ Res.

